# Molecular Dynamics Simulations Suggest Ligand’s Binding to Nicotinamidase/Pyrazinamidase

**DOI:** 10.1371/journal.pone.0039546

**Published:** 2012-06-26

**Authors:** Ji-Long Zhang, Qing-Chuan Zheng, Zheng-Qiang Li, Hong-Xing Zhang

**Affiliations:** 1 State Key Laboratory of Theoretical and Computational Chemistry, Institute of Theoretical Chemistry, Jilin University, Changchun, People’s Republic of China; 2 Key Laboratory for Molecular Enzymology and Engineering of the Ministry of Education, Jilin University, Changchun, People’s Republic of China; University of Westminster, United Kingdom

## Abstract

The research on the binding process of ligand to pyrazinamidase (PncA) is crucial for elucidating the inherent relationship between resistance of *Mycobacterium tuberculosis* and PncA’s activity. In the present study, molecular dynamics (MD) simulation methods were performed to investigate the unbinding process of nicotinamide (NAM) from two PncA enzymes, which is the reverse of the corresponding binding process. The calculated potential of mean force (PMF) based on the steered molecular dynamics (SMD) simulations sheds light on an optimal binding/unbinding pathway of the ligand. The comparative analyses between two PncAs clearly exhibit the consistency of the binding/unbinding pathway in the two enzymes, implying the universality of the pathway in all kinds of PncAs. Several important residues dominating the pathway were also determined by the calculation of interaction energies. The structural change of the proteins induced by NAM’s unbinding or binding shows the great extent interior motion in some homologous region adjacent to the active sites of the two PncAs. The structure comparison substantiates that this region should be very important for the ligand’s binding in all PncAs. Additionally, MD simulations also show that the coordination position of the ligand is displaced by one water molecule in the unliganded enzymes. These results could provide the more penetrating understanding of drug resistance of *M. tuberculosis* and be helpful for the development of new antituberculosis drugs.

## Introduction

**Figure 1:**
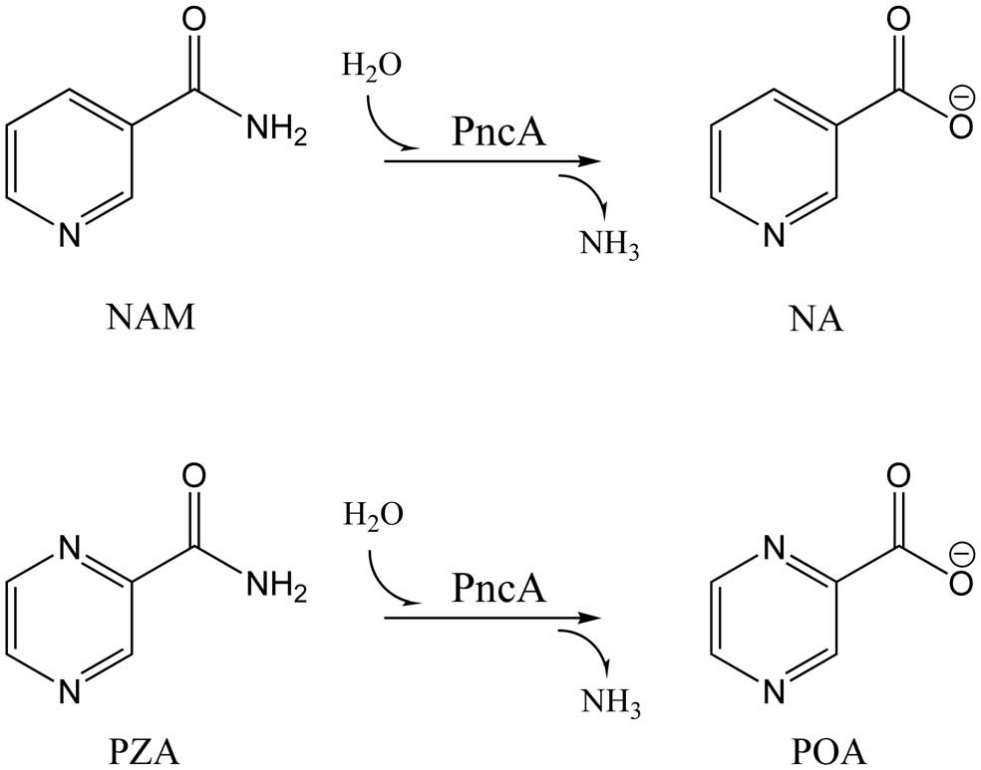
Hydrolysis reaction of NAM and PZA catalyzed by PncA.

Nicotinamidase is an important metabolic enzyme that can hydrolyze nicotinamide (NAM) to nicotinic acid (NA) (Figure 1) [1], always involved in the nicotinamide adenine dinucleotide (NAD^+^) salvage pathway of several pathogenic microorganisms such as *Borrelia burgdorferi* [2], *Brucella abortus* [3], and *Mycobacterium tuberculosis* [4]. Because of lacking a de novo NAD^+^ biosynthetic pathway like mammals [5], [6], many pathogens have to rely on the transformation from host’s NAM to their own NAD^+^ by nicotinamidase to keep the stabilization of NAD^+^ concentrations [2], [7]. Consequently, the nicotinamidase activity is of particular importance for the viability of these pathogens. Recently, targeting NAD^+^ biosynthesis as an antibiotic approach has been the intense subject of scientific investigation [7], [8]. Furthermore, the more important reason why nicotinamidase has attracted widespread attention is that the *Mycobacterium tuberculosis* nicotinamidase (*Mt*PncA) can convert the NAM analogue prodrug pyrazinamide (PZA) into the bacteriostatic compound pyrazinoic acid (POA) (Figure 1), hence the alternative name, pyrazinamidase (PncA) [9]–[12]. PZA, in combination with rifampicin and isoniazid, is the first-line drug recommended by the World Health Organization for the treatment of tuberculosis [10], [11], [13], [14]. The addition of PZA to this regimen leads to a significant reduction in the length of chemotherapy, allowing the conventional 9-month tuberculosis treatment to be shortened to 6 months [15]. The latest research has proved that the active form of PZA, POA, targets the ribosomal proteins S1 (RpsA) of *M. tuberculosis*, a vital protein in protein translation and the ribosome-sparing process of trans-translation, hence a subsequent inhibition of trans-translation and the shortened duration of tuberculosis chemotherapy [16]. But the binding of PZA to *Mt*PncA and activation by the enzyme are the essential prerequisites before functioning as the effective antituberculosis drug.

Despite the significance of PncA in the NAD^+^ salvage pathway and the PZA activation, its mechanism of action has not yet been fully understood. Since mutations in *pncA* gene were found by Scorpio and Zhang [17], a lot of researches have identified various mutations in *pncA* gene that can lead to the loss of PncA activity, which are thought to be highly correlated with the PZA resistance in *M. tuberculosis* [18]–[23]. One of the most striking features of these mutations is their diversity, with hundreds of mutations scattered throughout the *pncA* gene, not only in the active site. In order to interpret how the mutations result in the PZA resistance, the structure/function relationships of PncA need to be deeply comprehended. In 2001, Du and colleagues [24] established the unliganded crystal structure of PncA from *Pyrococcus horikoshii* (*Ph*PncA), which shows that *Ph*PncA has a Zn^2+^ ion coordinated by Asp52, His54 and His71, as well as a catalytic triad consist of Asp10, Lys94 and Cys133. Recently, the crystal structures of the *Acinetobacter baumanii* PncA (*Ab*PncA) with NA and POA bound at the active site were obtained in which the pyridine nitrogen of NA or POA was found to be directly tethered to a divalent metal cation (assumed to be Zn^2+^) [25]. In a more recent publication, French et al. [26] reported several high-resolution crystal structures of the nicotinamidase from *Streptococcus pneumoniae* (*Sp*Nic) in unliganded and ligand-bound forms, even with a trapped nicotinoyl thioester intermediates. These crystallography data have provided structural evidence of several proposed reaction intermediates and allowed for a more complete understanding of the reaction mechanism of PncA. Apart from the above-mentioned researches, which mainly focus on the catalytic mechanism and the active site residues, the latest crystallographic experiment has highlighted the underestimated importance of protein folding and thermal stability in the *Mt*PncA activity [27].

Indeed, the active-site residues of PncA play a key role in enzymatic catalysis and function, and should be responsible for the activity loss of numerous mutations. But not all the mutations inducing the activity loss occur in the active site [17]–[22], indicative of the significance of the non-active-site residues in PncA function. Among the residues, those in the binding pathway of ligand must be quite crucial for prompting the entrance of ligand to active site and proper orientation. However, a great number of the present researches on PncA pay less attention to the detailed binding process of ligand to PncA, which affirmably hinders the adequate understanding of PncA’s function. The further work of ligand’s binding or unbinding would be necessary for an in-depth illustration of the importance of those non-active-site residues. In this paper, the molecular dynamics (MD) simulation methods, which have been successfully applied to many similar researches [28]–[30], were used to explore the binding pathway of NAM to two PncA enzymes from two different bacteria (*Ab*PncA and *Sp*Nic). Based on the steered molecular dynamics (SMD) simulations, the potential of mean force (PMF) for three different pulling directions was constructed to determine the most possible binding or unbinding pathway. And then the role of some important residues in the pathway was proposed by calculation and analysis of the interaction energies between the ligand and two enzymes. Some shared structural characteristics of PncA family were presented by the superimposition and comparative analysis among several PncA proteins. Our present work would be helpful for the further research on PncAs and the development of new antituberculosis drugs.

## Results and Discussion

### Construction of Two Initial Models

Up to now, the crystal structure of NAM-bound *Ab*PncA has not been obtained in the experiment. But an analog of *Ab*PncA enzyme, *Sp*Nic, has been crystallized together with several different ligands, which involve not only NA but also NAM [26]. Superimposition and comparison of these *Sp*Nic-ligand complex structures show that in spite of different substituent groups, each ligand binds to the catalytic metal Zn^2+^ by the nitrogen atom of its respective pyridine ring. In addition, the proposed reaction mechanism of nicotinamidase also suggests that the coordination bond of pyridine ring to Zn^2+^ is always kept during the whole catalytic process [25], [26], [31]. Consequently, it is reasonable and reliable that NAM adopts the similar binding mode to NA in *Ab*PncA.

### Two Systems’ Stability in Conventional Molecular Dynamics Simulations

A total 13 ns free conventional MD simulation, including a 10 ns simulation in the NPT ensemble, was performed for each enzyme-ligand complex in order to relax the collision among some atoms and obtain a more stable conformation. The structural stability of two enzymes was assessed by calculation of the root-mean-square-deviation (rmsd) values. The rmsd values of all atoms and alpha carbon atoms (Cα) of two enzymes were calculated by taking their respective initial structures, i.e. crystal structures, as the reference points. The rmsd values, as the function of simulation time, demonstrate that the overall conformations of two proteins appear to be equilibrated in a very short time at the beginning of the free MD simulations (Figure S1 in the Supporting Information). The rmsd values of all atoms and Cα atoms of *Ab*PncA are generally stabilized near 1.8 and 1.1 Å, respectively, and the corresponding values of *Sp*Nic are 1.7 and 0.9 Å; accordingly, it can be inferred that the overall conformations of two proteins have been stable. But such the rmsd calculations have just estimated the global stability of the conformations and not considered the ligands and the local region of the proteins, especially the active site. In order to identify the local stability of the active site, several physical parameters, including some coordination bond lengths and dihedral angle values, were monitored with the simulation time at the NPT stages (Figure S2, S3, S4). The results indicate that the bond lengths of all the coordination bonds to the metal ion Zn^2+^ keep stable in the simulations and their average values, with the fluctuation of less than 10%, are very close to those average values in the experimental structures. Among these coordination bonds, those of NAM to Zn^2+^ in two proteins have the nearly equal values to the crystal structures (Figure S2). The sulfur atoms in the catalysis cysteine residues (Cys159 in *Ab*PncA and Cys136 in *Sp*Nic) have a bit longer distance to the carbonyl carbon atoms of NAM than those in the crystal structures (Figure S3), which may be affected by the protonation of the sulfhydryl groups in the cysteine residues. By the examination of the dihedral consisting of the coordination oxygen atom of Asp, Zn^2+^, the pyridine nitrogen atom and the carbonyl carbon atom of NAM, the pyridine ring plane of the ligand doesn’t display the rotation movement throughout the whole simulation process, despite a difference of about 7° from the experimental structures (Figure S4). All the check on the interaction details suggests that the active sites of two proteins are locally stable. In general, the global and local conformations of the two complexes both validate the stability at the free MD stages, which is really necessary for the sequential SMD simulations.

### Unbinding Pathway of Nicotinamide

**Figure 2:**
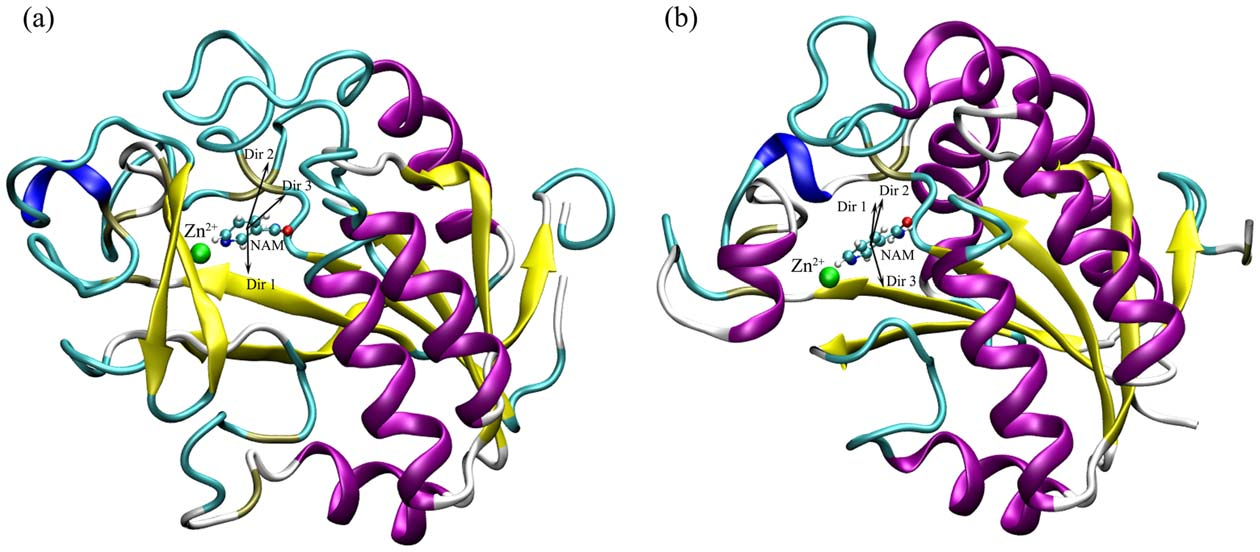
The pulling directions of NAM in two proteins: (a) *Ab*PncA and (b) *Sp*Nic during the SMD simulations.

**Figure 3:**
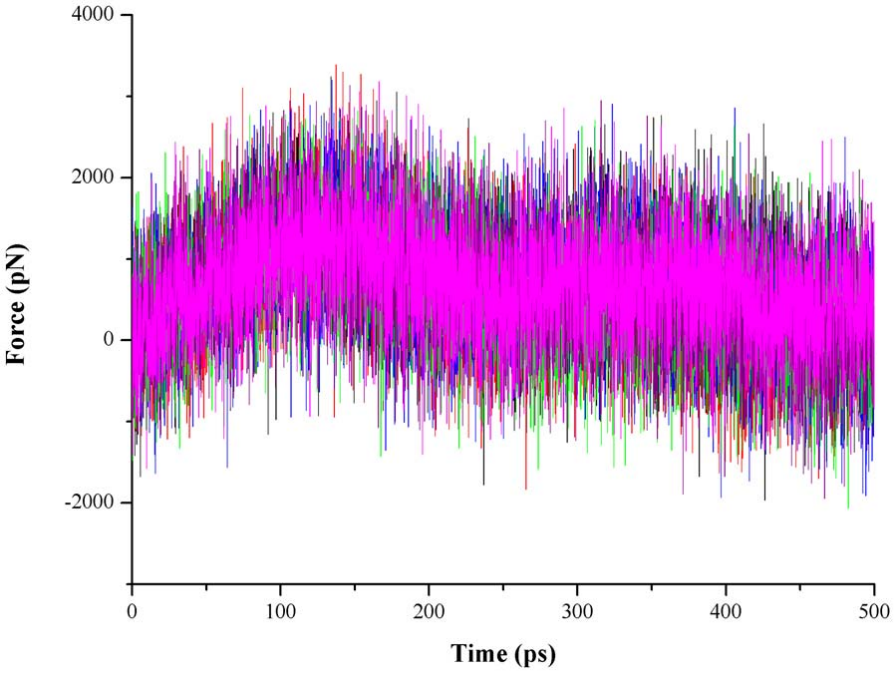
Six profiles of the applied forces along Direction 2 in *Ab*PncA.

The constant velocity SMD (cv-SMD) simulations were performed to pull the ligand NAM along the predetermined different directions. Through careful inspection of the NAM binding sites in two enzymes, as well as comparative analysis of the NAM-PncA interaction and the proposed catalytic mechanism [25], [26], three possible pathways were chosen to carry out the SMD simulations (Figure 2). In *Ab*PncA, three direction vectors are as follows: (1) Direction 1 is determined by the vector from Phe21 HE1 to NAM CE2 (the name in the force field); (2) Direction 2 is the vector from Wat2142 OH2 to NAM CE2; (3) Direction 3 is from Asp54 OD2 to NAM CE2 (Figure 2(a)). And in *Sp*Nic (Figure 2(b)), three directions are Direction 1 (the vector from Wat204 OH2 to NAM CE2), Direction 2 (the vector from Asp53 OD1 to NAM CE1) and Direction 3 (the vector from Asn72 OD1 to NAM NZ), in which Direction 1 and 2 are quite similar to each other but different from Direction 3 (Here, we chose two similar pathways in *Sp*Nic with the aim of validating the rationality of methods and evaluating the choice of pathways). The superimposition with the rmsd value of 2.3 Å of Cα atoms between two proteins indicates that Direction 1 in *Ab*PncA is similar to Direction 3 in *Sp*Nic, and Direction 2 in *Ab*PncA is similar to Direction 1 and 2 in *Sp*Nic. For each direction in two proteins, six SMD simulations were performed with the stiff spring constant. Figure 3 has just depicted the six curves of the pulling forces exerted on NAM along Direction 2 in *Ab*PncA. All the other curves also show the similar changing behavior. The large fluctuation of the applied forces in the pulling process results from the stiff spring constant, 10000 pN/Å. The large spring constant can ensure that the reaction coordinate follows closely the point to which it is constrained. It has been shown by the monitoring of the SMD trajectories that the displacement of the mass center of NAM has a simple linear relationship with the time axis. It can been found from Figure 3 that during the dissociation process of NAM, the exerted forces keep increasing in initial stage, and after reaching a maximum, the values of the pulling forces begin to drop down, implying the escape of the ligand from the bondage of the residues in the active site. However, some other residues in the pathway still have effect on the unbinding of NAM, and they will be analyzed in detail below.

**Figure 4:**
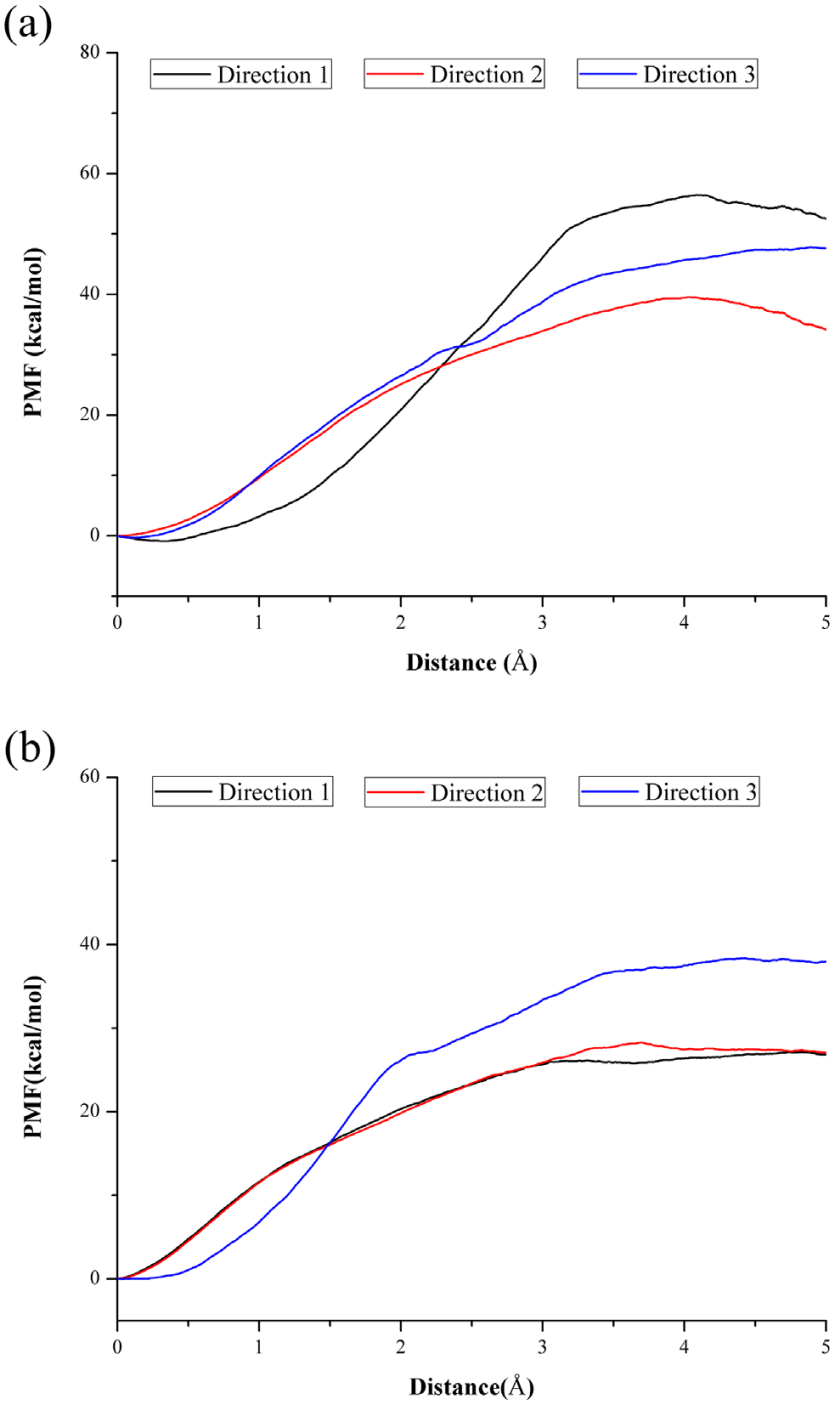
PMF profiles as a function of NAM’s displacement along the different directions in two proteins: (a) *Ab*PncA and (b) *Sp*Nic.

The potential of mean force (PMF) was constructed from six repeated cv-SMD simulations for each of the predefined directions, in which the direction with the lowest energy barrier can be regarded as the most possible unbinding or binding pathway. The calculated PMF profiles were recorded in Figure 4. It can be observed from Figure 4(a) that in the pathway along Direction 2 there is the lowest free energy barrier, about 40 kcal/mol, and the pathways along Direction 1 and 3 are both energetically unfavorable for the ligand’s dissociation from *Ab*PncA. So the pathway along Direction 2 is the most possible pathway for ligand’s unbinding, of course also for its binding. In *Sp*Nic (Figure 4(b)), due to the similarity between Direction 1 and 2, two PMF curves coincide well with each other and their energy barriers, around 27 kcal/mol along Direction 1, are lower than that in the pathway along Direction 3. Thus the pathway along Direction 1 or 2 are more favorable for NAM’s unbinding or binding. As mentioned above, the superimposition of two proteins proves that Direction 2 in *Ab*PncA corresponds to Direction 1 and 2 in *Sp*Nic; on the other hand, for two proteins, their respective lowest energy barriers exist in the pathways along these two similar directions. Therefore, such the pathway could be their general unbinding/binding pathway. Further, due to the structural similarity of all kinds of nicotinamidases, it is speculated that the above-mentioned pathway also applies to the other enzymes. In spite of the lowest energy barriers in each protein, two minima (40 vs. 27 kcal/mol) have the difference of 13 kcal/mol, which is ascribed to the different position of the active sites in two proteins. In comparison, the active site in *Ab*PncA is buried in the interior of the protein while that in *Sp*Nic is close to the surface of the protein. Therefore the ligand in *Sp*Nic is susceptible to the external environment, such as solvent, and its combination with the protein is weaker than that with *Ab*PncA, which results in the lower free energy barrier.

### Important Residues Affecting Ligand’s Unbinding or Binding

Based on the optimal pathways determined above, many important residues affecting the binding/unbinding of NAM can be explained by the calculations of interaction energies between the ligand and the proteins. In the ligand’s dissociation process, the analyses of the total interaction energy between NAM and two proteins reveal that the van der Waals (Vdw) interaction is dominant relative to the electrostatic interaction, which is in good agreement with the observation that the unbinding pathways in two proteins mainly consist of the hydrophobic residues. It also suggests that the aromatic ring of the ligand dominates the binding/unbinding process, so the improvement or design of drug with more hydrophilic substitutional groups may decrease its binding activity with the target proteins in spite of possible higher affinity in the final complex states.

**Figure 5:**
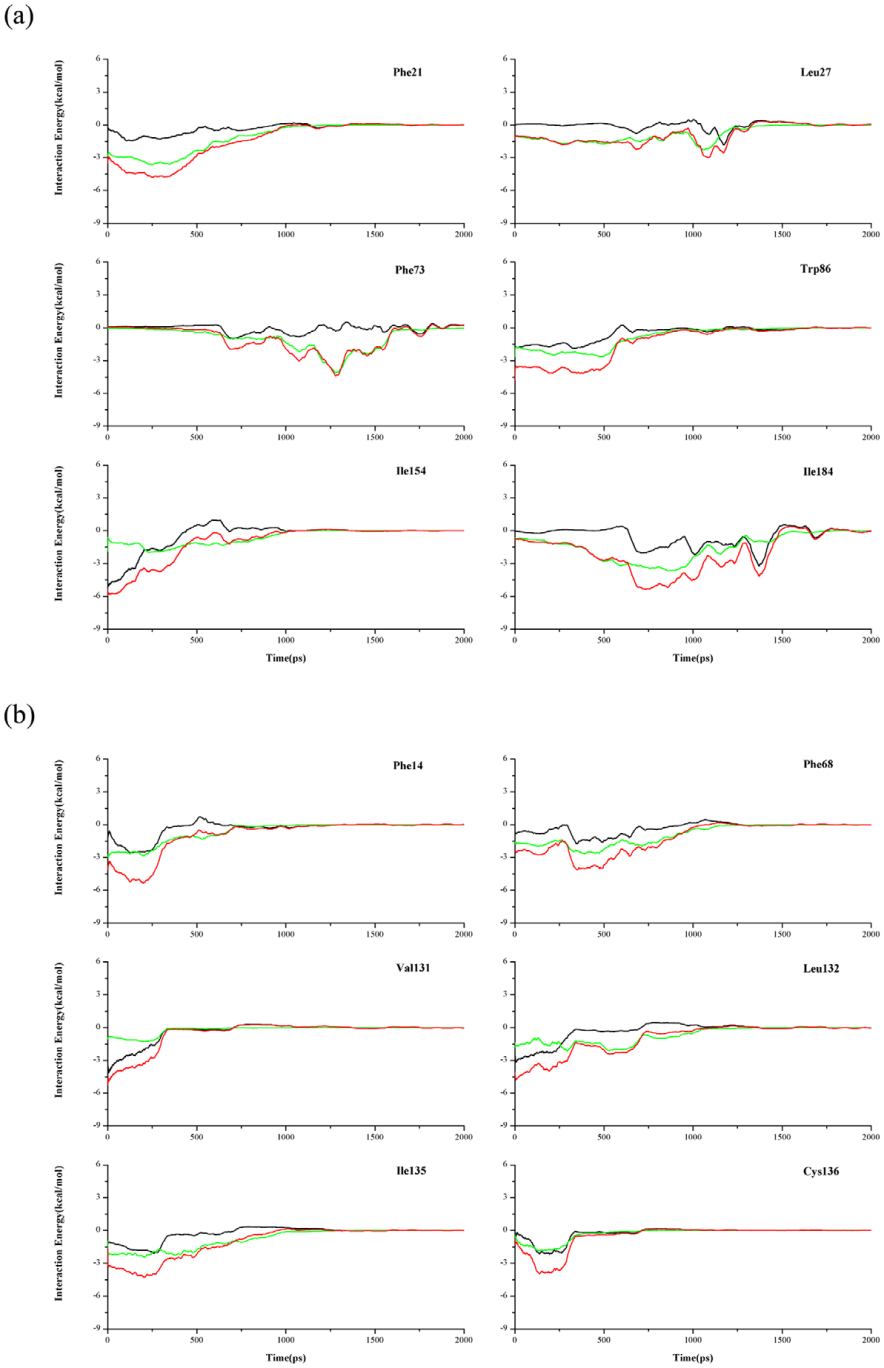
Time dependence of all kinds of interaction energies between NAM and some related residues of two proteins: (a) *Ab*PncA and (b) *Sp*Nic during the SMD simulations. The Vdw interaction energies are colored in green, the electrostatic interaction energies in black and the total interaction energies in red. The curves of the interaction energies represent the smoothed values from the FFT smoothing method of 50 ps.

It is obvious that the different residues in the possible binding/unbinding pathways have different effects on the interaction of NAM with two proteins. And the interaction energies between the ligand and all the important residues have been calculated and presented in Figure 5, in which the electrostatic interaction energies are marked as the black curves, the Vdw interaction energies are the green curves, and the total interaction energies are the red ones. The total interaction energies between these residues and NAM all possess the minimum lower than −3 kcal/mol, and display better consistency among six SMD trajectories. Notice that the main interaction of NAM with most residues is the Vdw interaction, which keeps consistent with the above results. In *Ab*PncA, a special residue is Ile154. The interaction energy between the residue and NAM gradually decreases with the displacement of NAM. Besides, the residue mainly interacts with the ligand by the electrostatic force. By the inspection of the SMD simulation trajectories, it is found that a hydrogen bond is formed between the main-chain carbonyl group of the residue and the amide group of NAM, and the side chain of the residue is relatively far from NAM. The oxyanion hole, consisting of two main-chain amides of *cis*-Ala155 and Cys159, also interacts with NAM by the electrostatic force, whereas the interaction of NAM with the two residues is not as strong as that with Ile154. In Figure 5(a), the other residues, except Ile154, display the minima during the unbinding process of the ligand, which play the impeditive role in the dissociation of NAM. Among them, the minimum for Phe21 appears at the beginning, and those for Leu27, Phe73 and Ile184 emerge at the latter stage, which is dependent on their positions in the dissociation pathway. The interaction between Trp86 and NAM stabilizes near the lower value in a longer time, which results from the relatively large side chain size of Trp86. Some other residues, including Val29, Tyr123 and Phe158, have the modest effect on the dissociation of NAM, with the total interaction energies more than −2.5 kcal/mol, though the residue Val29 also hinders the ligand’s unbinding or binding.

The structural superimposition demonstrates that the residues Phe14, Phe68, and Val131 in *Sp*Nic correspond to the residues Phe21, Trp86, and Ile154 in *Ab*PncA, respectively. The changing trend of their interaction curves is also very similar. In comparison with their counterpoint residues Ala155 and Phe158 of *Ab*PncA, the residues Leu132 and Ile135 of *Sp*Nic have the large interaction energy with NAM, indicative of the stable Vdw interaction and unstable electrostatic interaction. The catalysis residue Cys136 is distinct from the corresponding residue Cys159 in *Ab*PncA. In *Sp*Nic, the stronger interaction comes up between Cys136 and NAM, which is attributed to the double contribution of both the electrostatic and Vdw interaction. The residue Leu21 displays large fluctuation of interaction energies in different SMD simulations. It may be caused by the exposure of the active site in *Sp*Nic. Leu21 in *Sp*Nic apparently lacks the restraints from the other residues, and features the larger degree of freedom. So the residue has the great possibility of the side chain rotating during the dissociation process of NAM.

### Protein’s Changes after Ligand’s Dissociation

**Figure 6:**
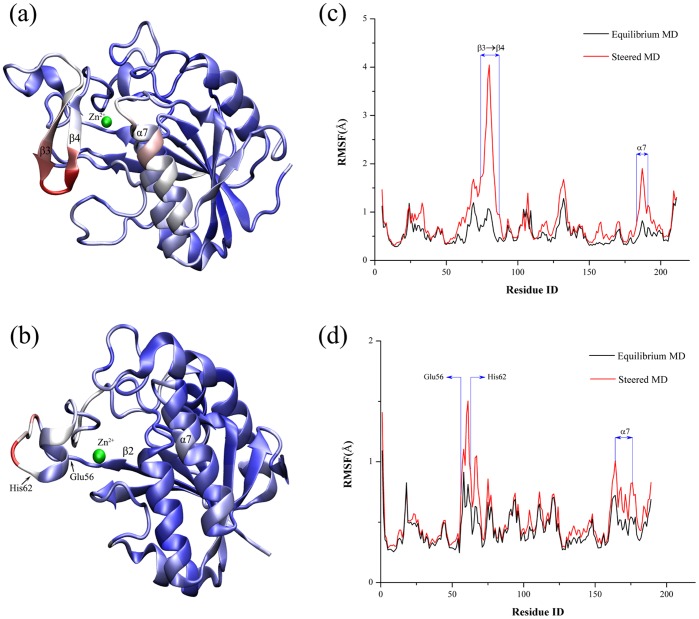
Conformational change of two proteins: (a) *Ab*PncA and (b) *Sp*Nic together with the corresponding rmsf values of their Cα atoms in the unbinding process of NAM.

During the binding or unbinding process, the interaction between NAM and the related residues of the proteins possibly leads to some changes of the proteins’ structures. Herein we have calculated the root-mean-square-fluctuation (rmsf) of the Cα atoms of two proteins in the SMD simulations and illustrated the relative motion in the interior of two proteins (Figure 6). Figure 6(a) describes the conformation’s change of *Ab*PncA in the dissociation of the ligand. As seen in the figure, some parts of the protein have produced the obvious changes as the ligand’s unbinding, which mainly concentrate the region between β3 and β4 sheets and a portion of α7 helix, surrounding the exit of the unbinding pathway of NAM. Such the motion is also reflected in the rmsf figure of the Cα atoms, in which the β3 to β4 parts of the protein have the largest rmsf values, and secondly, the α7 helix also possesses the considerable fluctuation. By careful investigation on the SMD trajectories, the β3 to β4 portion of *Ab*PncA is observed to behave like a “gate”, that is, when a ligand leaves from the active site, the gate opens to the external environment, and in contrast when a ligand is bound to the protein, the gate is closed to lessen the exoteric effect on the ligand’s binding and protein’s catalysis. However, in *Sp*Nic there is no similar portion to that “gate” in *Ab*PncA. In *Sp*Nic, both the internal motion of protein and the rmsf values of the Cα atoms show that the biggest conformational change of the protein takes place in the region between Glu56 and His62 and the whole α7 helix just has the slight fluctuation (Figure 6(b)). Owing to the absence of the “gate” region, the active site in *Sp*Nic is exposed to the external environment, so the ligand’s binding to the protein is liable to be disrupted by the solvent. Hence such the binding is weaker than that in *Ab*PncA, and is easy to break, which is consistent with the PMF results above-mentioned.

**Figure 7:**
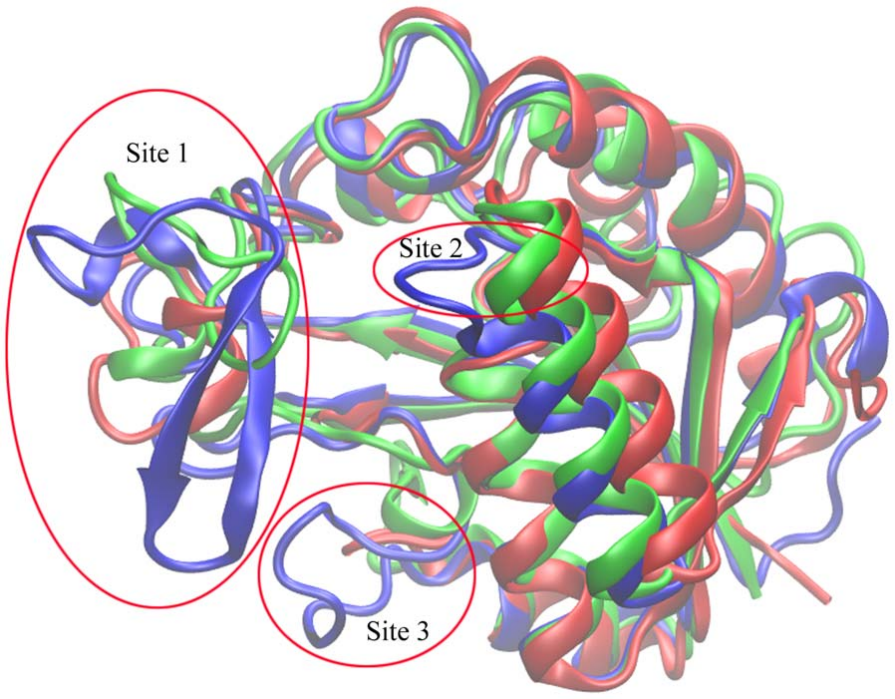
Superimposition and structural comparison of three proteins: *Ab*PncA (blue), *Sp*Nic (red) and *Mt*PncA (green).

A recently obtained X-ray crystal structure of PncA from *Mycobacterium tuberculosis* (*Mt*PncA) provides us with a chance of aligning the protein with the present-researched two proteins and performing the comparative analysis [27]. Figure 7 shows the structural alignment of three proteins *Ab*PncA (blue), *Sp*Nic (red) and *Mt*PncA (green) from *Acinetobacter baumanii*, *Streptococcus pneumoniae* and *Mycobacterium tuberculosis*, respectively. Generally speaking, the structure of *Ab*PncA has the noticeable difference from that of the other two proteins while *Sp*Nic and *Mt*PncA have the quite similar spatial structures. Just as mentioned above, there is a “gate” region in Site 1 of *Ab*PncA, while there is no such the region in the corresponding site of *Sp*Nic that the protein can’t bind its substrate as tightly as *Ab*PncA. For *Mt*PncA, its Site 1 region looks like neither that of *Ab*PncA nor that of *Sp*Nic. This site in *Mt*PncA is composed of a loop region (Ile52-Trp68) with greater flexibility, which is just located near the exit of ligand. Accordingly, this part of *Mt*PncA is thought to produce the bigger movement in the process of ligand’s binding or unbinding and thus directly affect the binding activity and catalysis of substrate. In fact, a particular metal-coordinated residue, His57, is just in the region. And the recent research [27] has also highlighted the importance of this region. On the other hand, owing to the absence of β-sheet like that in *Ab*PncA, *Mt*PncA may not bind substrate as strongly as *Ab*PncA. In Site 2 region marked in Figure 7, it can be obviously seen that the conformation of *Ab*PncA has some significant distinctions from that of the other two proteins. The Site 2 region of *Ab*PncA is fairly close to the binding/unbinding pathway of ligand, which is the reason for its fluctuating. And the corresponding parts of *Mt*PncA and *Sp*Nic are alike. According to the analysis of *Sp*Nic, the region of *Mt*PncA will also be stable. In spite of the different spatial conformation of three proteins in Site 3 region as described in Figure 7, on the basis of the investigation into the other two proteins, it is speculated that the region of *Mt*PncA would keep the conformational stability during the binding/unbinding process of ligand.

**Figure 8:**
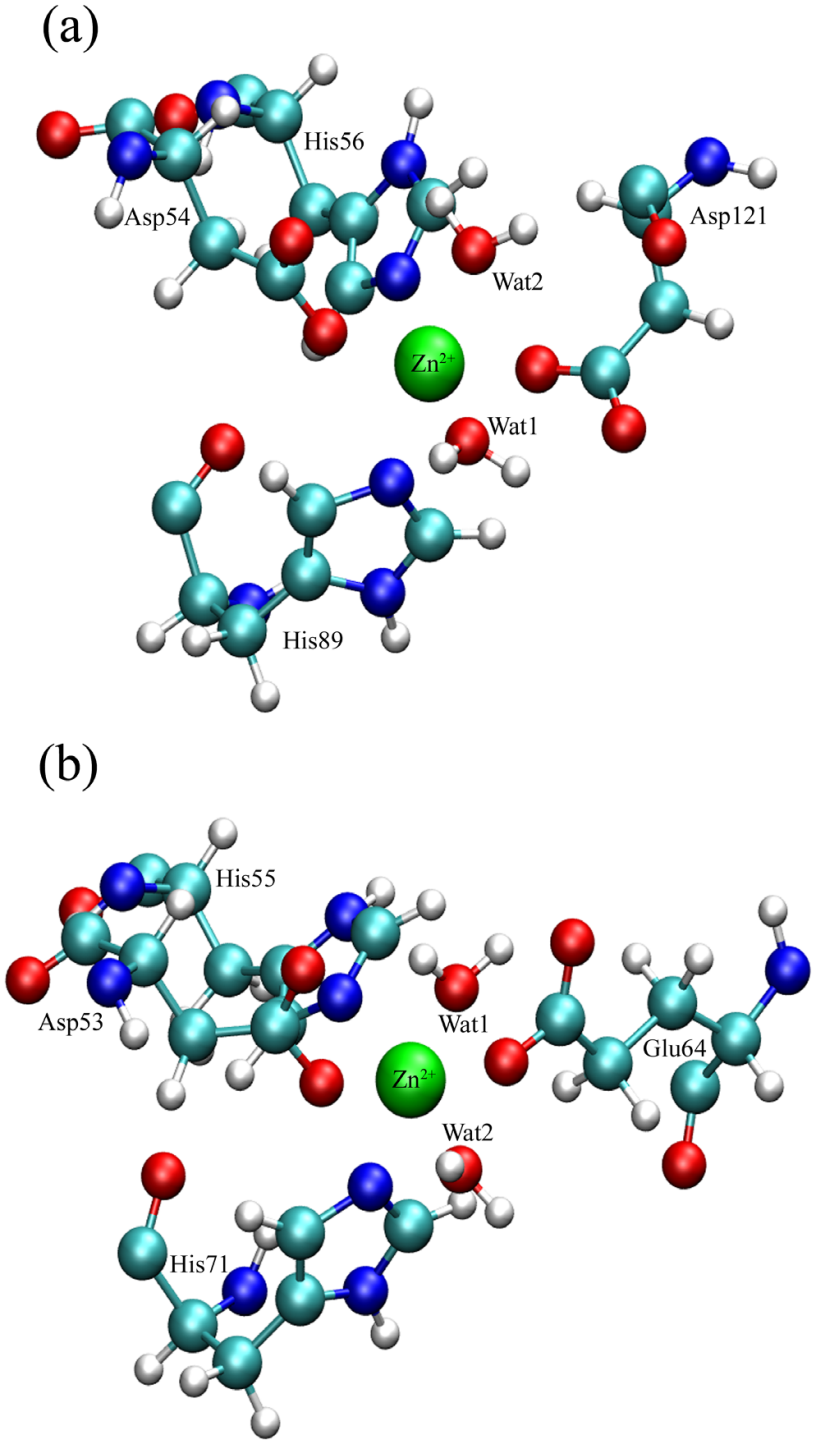
Conformations of the active site in two proteins: (a) *Ab*PncA and (b) *Sp*Nic after the dissociation of NAM.

Because of the direct coordination of the ligand NAM to the metal center, the going of the ligand from the active site of the proteins will induce the change of the metal ion coordination, in addition to the transformation of the proteins’ conformation (Figure 8). The details on the change process were found by the careful inspection of the SMD trajectories. After the ligand departs from the metal ion Zn^2+^ in the active site of *Ab*PncA, its coordination position empties, which turns the metal ion into a penta-coordinated center. The case is energetically unstable. Then one of the coordinated waters moves to the coordination position of the ligand. Owing to the H-bond interaction between the coordinated water molecules and Asp121, the residue is pulled towards the metal ion by the locomotive water. The result is that Asp121 replaces the shifted water to coordinate with the metal ion. The other coordination residues still stay in their original places. So Zn^2+^ in the active site of *Ab*PncA restores to the hexa-coordinated structure (Figure 8(a)). The same thing happens to *Sp*Nic. In the active site of *Sp*Nic, a residue Glu64, instead of the coordinated water in *Ab*PncA, directly coordinates to the metal ion. After the ligand’s leaving, one water molecule from the external solvent comes in and thus takes up the coordination position of NAM. And the other coordination of Zn^2+^ remains unchanged (Figure 8(b)). By comparison, the active site configurations of two proteins are very similar at the time of the absence of the ligand (Figure 8). Here, the research on the ligand’s unbinding from protein provides some clues about the binding of ligand to the proteins because these two processes are quite the reverse.

### Conclusions

In the present study, NAM, the analog of antituberculosis prodrug PZA, were chosen as a probe to investigate the ligand’s binding to the two PncA enzymes, *Ab*PncA and *Sp*Nic. After the long MD simulations, the PMF’s calculations along the different unbinding directions were carried out on the basis of the SMD simulations to determine the most possible binding pathway of the substrate. The superimposition and comparison of the proteins show that the determined binding pathways, with the lowest free energy barriers, in two PncAs share much the same direction. Further, owing to the structural similarity of all kinds of PncAs, it is speculated that such the pathway should be the general binding or unbinding pathway for all PncAs. After the ascertainment of the most possible binding pathway, the calculations of interaction energies shed light on some important residues affecting the binding of ligand to PncA, which contain not only the active-site residues but also the non-active-site ones. The unbinding or binding of ligand also affects the structure of PncA. Two PncAs chosen in our research both exhibit the greater extent relative motion in the same region in the unbinding process of ligand. The consistency of structural change in *Ab*PncA and *Sp*Nic give us a hint that the corresponding regions in the other PncAs will also show the similar interior motion. The importance of the determined regions for *Mt*PncA has been highlighted by the recent research [27]. Moreover, after the unbinding of ligand, the active sites of two unliganded enzymes also display some similar changes, namely, the coordination positions of NAM are taken up by a water molecule. One special thing for *Ab*PncA is that the residue Asp121 near the active site coordinates with the metal ion Zn^2+^ after NAM departs. Our work could provide penetrated insight into drug resistance of *M. tuberculosis* and would contribute to the guideline for the new antituberculosis drug design.

## Materials and Methods

Preparation of initial crystal structures were performed with the Discovery Studio 2.5 software package [32] on a Dell Precision WorkStation T5400. The conventional MD and SMD simulations were carried out on the Inspur Cluster with the parallel MD program NAMD 2.6 [33]. Topology and force field parameters were assigned from the CHARMM27 protein lipid parameter set [34] for the proteins and from the CHARMM General Force Field (CGenFF) parameter set [35] for the small molecule ligand NAM.

### Preparation of Initial Models

The initial models of MD simulations came from two X-ray crystal structures: (i) NA-bound *Ab*PncA (PDB ID: 2WT9) and (ii) NAM-bound *Sp*Nic (PDB ID: 3O94) [25], [26]. Both of the structures contain more than one chain. In order to decrease the system size, only chain A was retained to carry out the MD calculations. The superimposition and comparison of several structures from *Sp*Nic show that PncAs with NAM, NA and nicotinoyl thioester are nearly same as each other in the active site conformation. Based on the inspection, the NAM-bound *Ab*PncA may also be considered to be same as the NA-bound one. So the NA ligand in 2WT9 was manually modified to be NAM by Discovery Studio Visualizer [32]. Three glycerin molecules were deleted in this pdb file. Besides, the C136S mutation in 3O94 was also restored to the normal Cys residue. Except the modifications above, no other change was introduced in order to as much as possibly preserve the initial structure.

### Conventional Molecular Dynamics

The above-prepared final structures were used to perform the conventional MD simulations. The missing atoms were added by means of the VMD program [36]. TIP3P water model [37], with a cubic box extending up to 10 Å from the solute in each direction, was used to solvate the whole protein-ligand complex. According to the physiological concentration, 154 mM, the corresponding Na^+^ and Cl^−^ ions were added to ensure the overall neutrality of the system.

Periodic boundary conditions were applied to the system to obtain consistent behavior. The cutoff distance of electrostatic interaction was set to 12 Å. And the electrostatic interaction between 1–4 atoms is taken into account. Van der Waals interactions were gradually turned off at a distance between 12 and 14 Å. The particle mesh Ewald (PME) method [38], [39] was employed every one step for the computation of long-range electrostatic forces. The pair list distance was 15.5 Å. The non-bonded pair list was updated every 20 steps. An integration time step of 1 fs was assumed. The whole system was first energy minimized with 10000 steps of conjugate gradients, by keeping the coordinates of protein backbone atoms and ligands restrained with a spring constant of 20 kcal mol^−1^ Å^−2^. After minimization, at the same restraint, the temperature of the system was gradually increased with Langevin dynamics [40] from 100 to 300 K by stepwise reassignment of velocities every 2 ps. Then another 1 ns MD simulation was performed, in which the constraint was gradually decreased to 0 with a step length of 4 kcal mol^−1^ Å^−2^. And then an additional 3 ns simulation was carried out without any constraint in the NVT ensemble. Following that, the pressure of the system was coupled to a reference pressure of 1 bar with a modified Nosé-Hoover Langevin piston method [41]. In the NPT ensemble, a total 10 ns MD simulation was implemented on the whole system to ensure the accomplishment of the equilibrium. The final equilibrium state was used for a restart point for further SMD.

### Steered Molecular Dynamics Simulation

In the SMD simulations, mimicking the AFM experiment, time-dependent external forces are applied to ligand to facilitate its unbinding from receptor, which usually could not be achieved by the conventional MD simulations. In the NAMD implement, SMD may be carried out with either a constant force applied to an atom (or set of atoms) or by attaching a harmonic (spring-like) restraint to one or more atoms in the system and then varying either the stiffness of the restraint or the position of the restraint [33]. The two kinds of simulations correspond to constant force (cf) SMD and constant velocity (cv) SMD, respectively. In the present study, the cv-SMD was adopted to calculate a so-called potential of mean force (PMF) using Jarzynski’s identity [42], [43]. In the cv-SMD simulations, the pulling velocity was set to 0.01 Å/ps, which has been used for the construction of PMF profiles [44]. A large spring constant k (k = 10000 pN/Å) was used to ensure that the pulled atoms follow the constraint closely, a condition known as the stiff spring approximation and required for an efficient application of Jarzynski’s identity [44], [45]. When performing the pulling simulations, the Zn^2+^ ion, together with the Cα atoms of its coordination residues, was kept to fix. An external steering force was applied on the mass center of ligand along the pre-defined directions (see Results and Discussion section). The pulling force experienced by the reference point was calculated by employing eq 1:
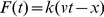
(1)where *k* is the force constant for pulling spring and *x* is the displacement of reference point from its original position. During the SMD simulations, the steering force was only applied along the pulling direction. The ligand was free from constraint in the plane perpendicular to pulling direction. The trajectories were saved for every 0.1 ps, and steering forces were recorded for every 20 fs. The SMD simulation for each pulling direction was repeated for six times with the same starting structure and the different random seeds.

### PMF Calculation Based on Jarzynski’s Equality

The free energy change along a reaction coordinate, which is termed the potential of mean force (PMF), was calculated by Jarzynski’s Equality on the basis of the repeated SMD simulations. Jarzynski’s Equality establishes a connection between equilibrium free energies calculation and nonequilibrium work output, and allows us to calculate PMF under nonequilibrium processes such as SMD simulations [42], [44]–[48]. The formula of Jarzynski’s Equality is as follow:
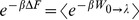
(2)where Δ*F* represents the free energy change between the initial and final states, *W_0→λ_* is the work performed from state 0 to state λ in the SMD simulation, and *β* = (*k_B_T*)^−1^, where *k_B_* and *T* are the Boltzmann constant and temperature, respectively. When the pulling speed in a SMD simulation is low enough that the process can be thought to be reversible, the work of the applied force is equal to the free energy difference of the system between the initial and final states. However, because of limited computing power, such a process is often simulated at a speed several orders of magnitude higher than the quasi-static speed, and besides simulations can sample only a small number of trajectories. In such a case, the exponential average term in broken brackets on the right-hand side of formula (2) is difficult to estimate as a result of large statistical errors caused by insufficient sample data. To prevent this problem, some necessary approximation methods are introduced, one of which is the expansion of Jarzynski’s Equality by cumulant expansion as follows:



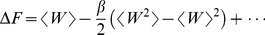
(3)If the distribution of work *W* shows Gaussian, the third- and higher-order cumulants are identically zero and the second-order cumulant expansion formula can be utilized to suppress statistical error [44]. Therefore the present PMF calculations were carried out by the second-order cumulant expansion formula. The estimators based on Jarzynski’s equality were used for estimating the convergence of the free energy [44].

## Supporting Information

Figure S1 RMSD profiles for all atoms and Cα atoms of two PncAs: (a) *Ab*PncA and (b) *Sp*Nic with respect to their respective reference structures (crystal structures) in the 13 ns free MD simulations.
(TIF)

Figure S2 Time dependence of all the Zn-coordinated bond lengths in the active sites of two PncAs: (a) *Ab*PncA and (b) *Sp*Nic in the 10 ns NPT MD simulations. The straight lines in the figure represent the corresponding values of those bond lengths in the experimental structures, i.e. the crystal structures.
(TIF)

Figure S3 Fluctuations of the distances between NAM carbonyl C atoms and the catalytic cysteine S atoms (Cys159 in *Ab*PncA and Cys136 in *Sp*Nic) in two PncAs: (a) *Ab*PncA and (b) *Sp*Nic during the 10 ns NPT MD simulations.
(TIF)

Figure S4 Change plots of a related dihedral in the active sites of two PncAs: (a) *Ab*PncA and (b) *Sp*Nic during the 10 ns NPT MD simulations. The dihedral consists of the following four atoms: the coordination O atoms of Asp residue (Asp54 in *Ab*PncA and Asp53 in *Sp*Nic), the metal Zn ion, the pyridine N atom of NAM, and the carbonyl C atom of NAM.
(TIF)
