# Supplementary figures and images for: Molecular Dynamics Simulations Suggest Ligand’s Binding to Nicotinamidase/Pyrazinamidase
Source: PLoS One. 2012 Jun 26;7(6):e39546. doi: 10.1371/journal.pone.0039546 (PMC3383691; doi:10.1371/journal.pone.0039546)

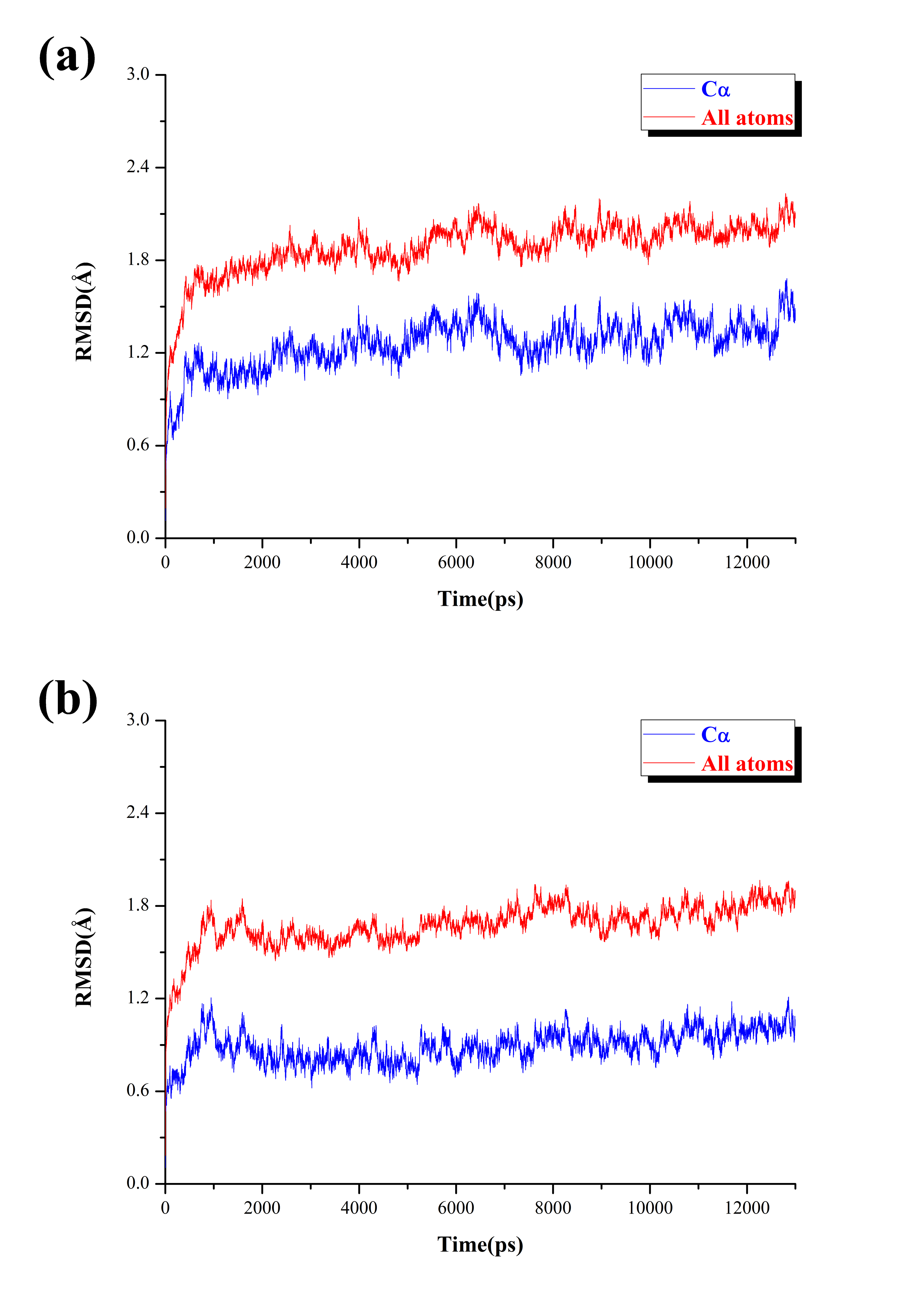

Supplement: Figure S1 — RMSD profiles for all atoms and Cα atoms of two PncAs: (a) AbPncA and (b) SpNic with respect to their respective reference structures (crystal structures) in the 13 ns free MD simulations. (TIF) [file pone.0039546.s001.tif]

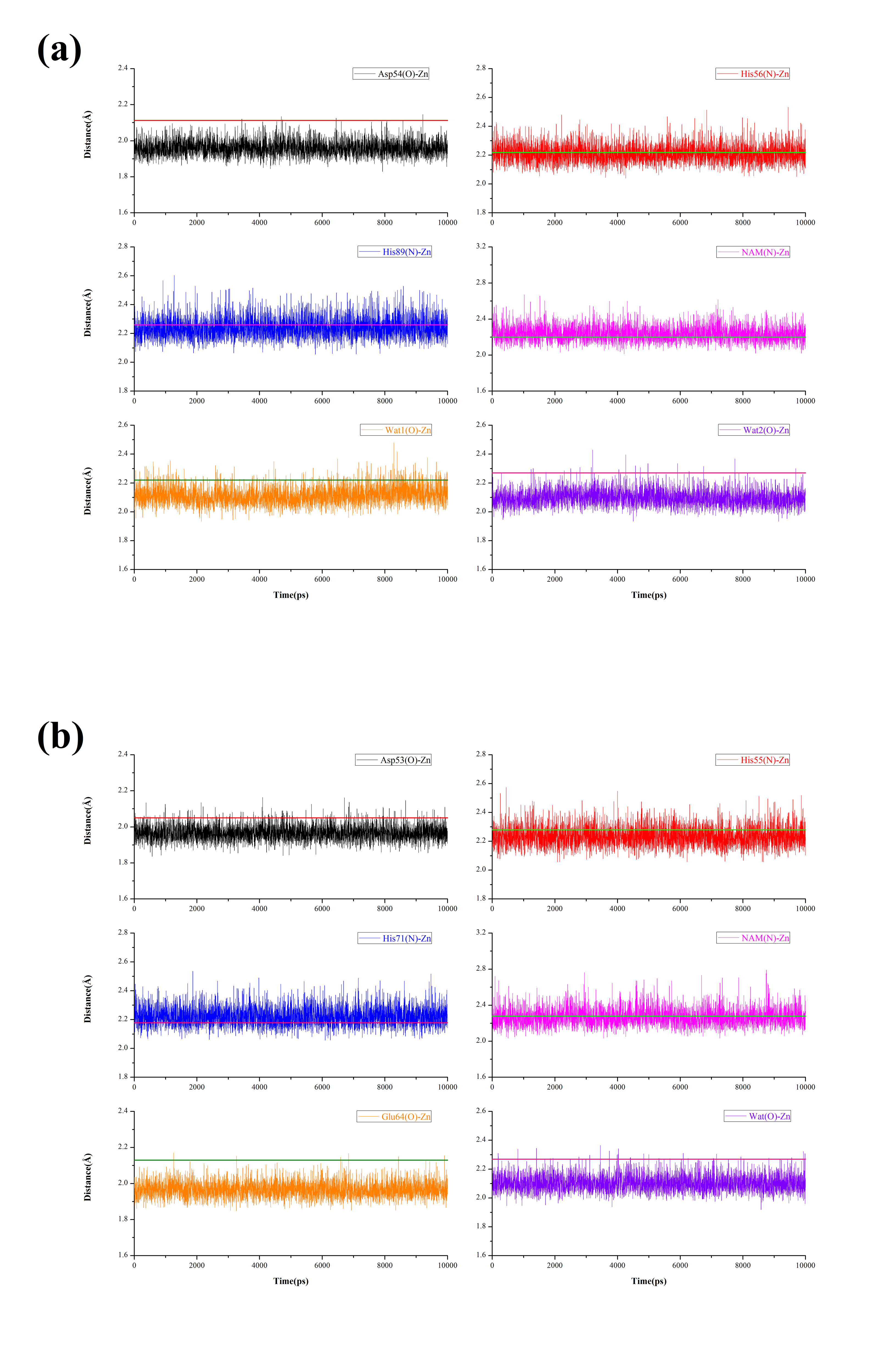

Supplement: Figure S2 — Time dependence of all the Zn-coordinated bond lengths in the active sites of two PncAs: (a) AbPncA and (b) SpNic in the 10 ns NPT MD simulations. The straight lines in the figure represent the corresponding values of those bond lengths in the experimental structures, i.e. the crystal structures. (TIF) [file pone.0039546.s002.tif]

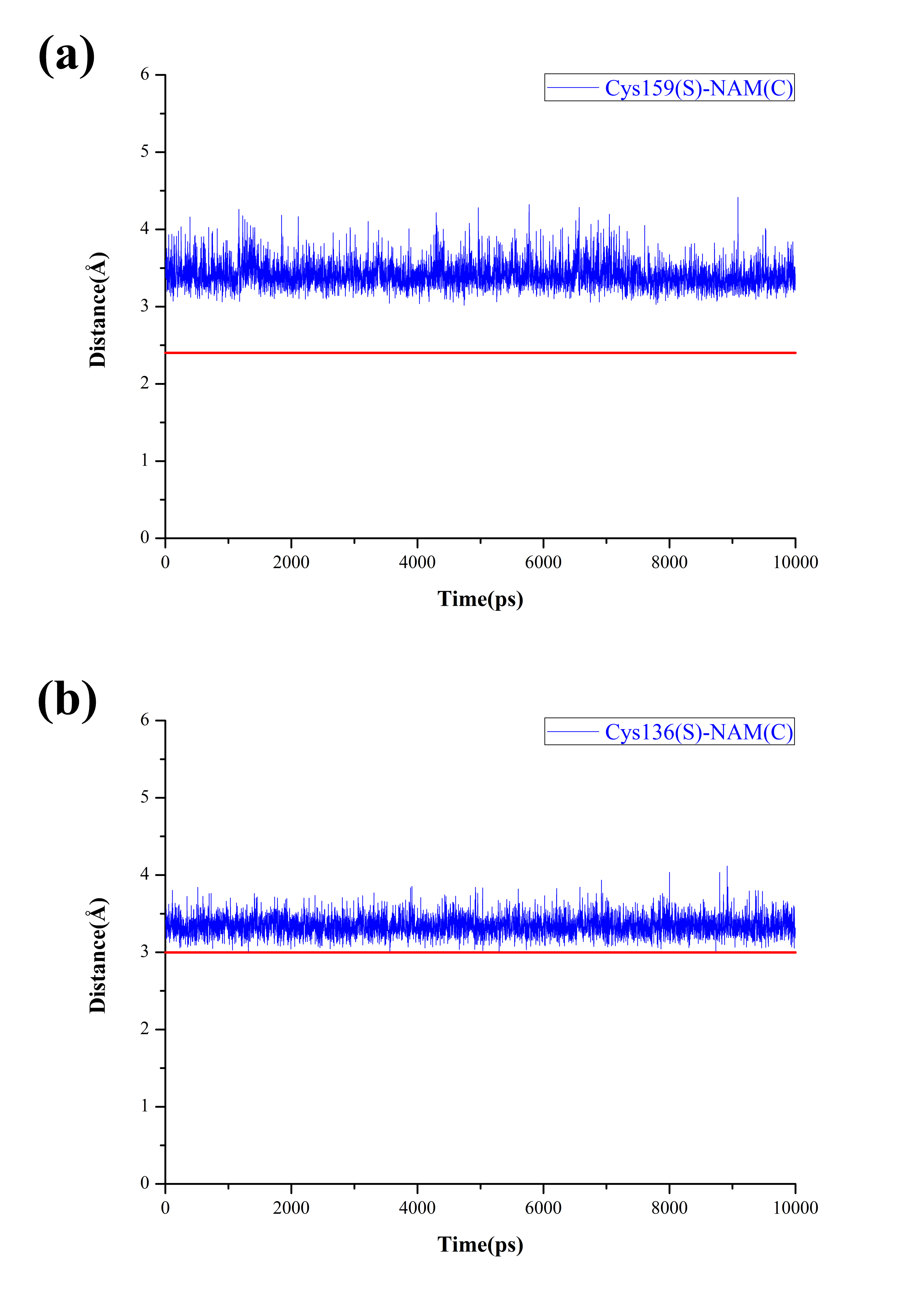

Supplement: Figure S3 — Fluctuations of the distances between NAM carbonyl C atoms and the catalytic cysteine S atoms (Cys159 in AbPncA and Cys136 in SpNic) in two PncAs: (a) AbPncA and (b) SpNic during the 10 ns NPT MD simulations. (TIF) [file pone.0039546.s003.tif]

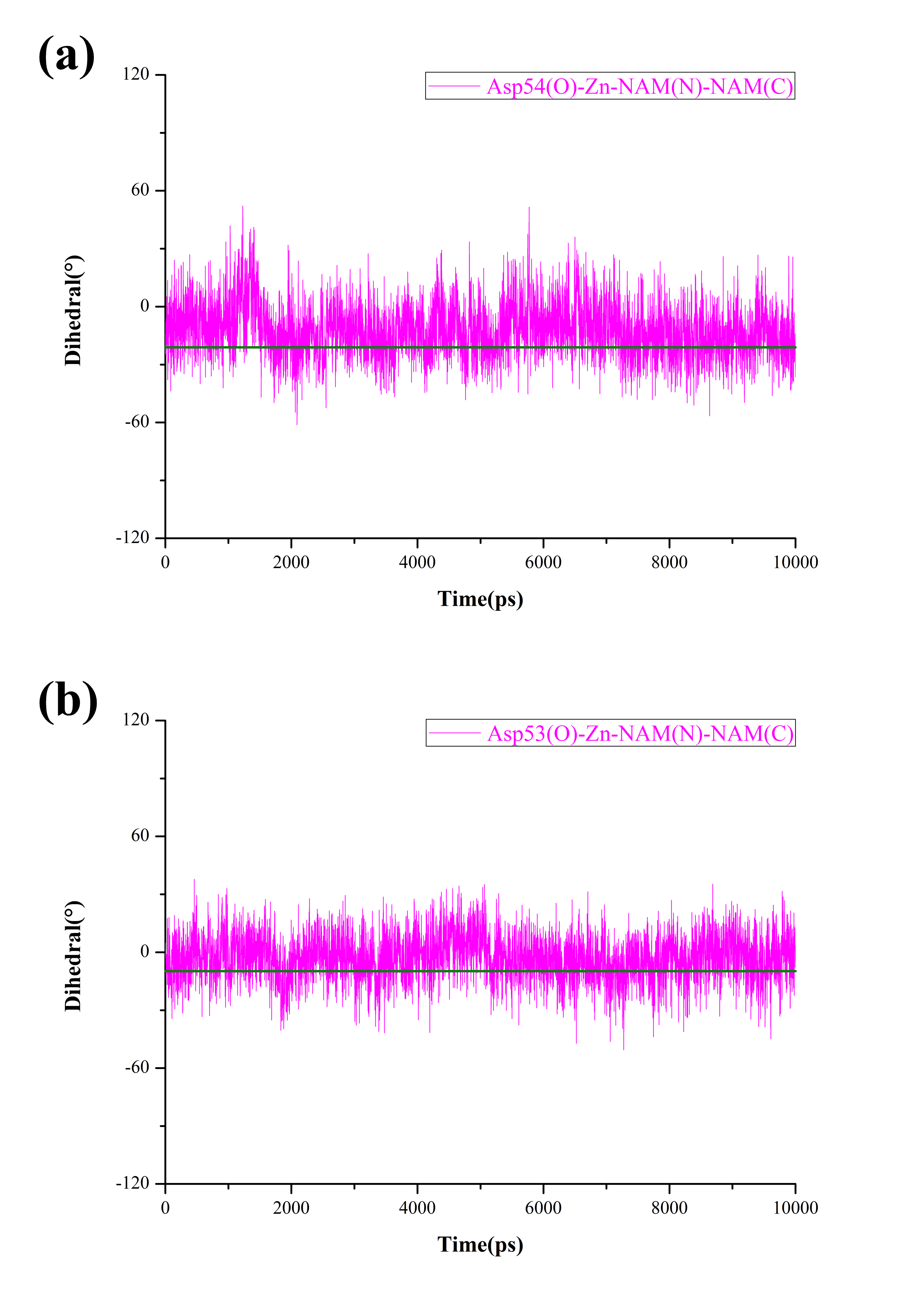

Supplement: Figure S4 — Change plots of a related dihedral in the active sites of two PncAs: (a) AbPncA and (b) SpNic during the 10 ns NPT MD simulations. The dihedral consists of the following four atoms: the coordination O atoms of Asp residue (Asp54 in AbPncA and Asp53 in SpNic), the metal Zn ion, the pyridine N atom of NAM, and the carbonyl C atom of NAM. (TIF) [file pone.0039546.s004.tif]
